# Beyond NT-proBNP and troponin: How machine learning redefines light-chain cardiac amyloidosis risk assessment

**DOI:** 10.1186/s12911-025-03207-0

**Published:** 2025-10-09

**Authors:** Danni Wu, Xiaohang Liu, Xinhao Li, Jia Chen, Xue Lin, Ligang Fang, Wei Chen

**Affiliations:** 1 Department of Integrative Medicine Cardiology, China-Japan Friendship Hospital (Institute of Clinical Medical Sciences), Chinese Academy of Medical Sciences & Peking Union Medical College, Beijing, 100730 China; 2 Department of Cardiology, Peking Union Medical College Hospital, Chinese Academy of Medical Sciences & Peking Union Medical College, Beijing, 100730 China; 3 Institute of Hematology & Blood Diseases Hospital, National Clinical Research Center for Blood Diseases, Chinese Academy of Medical Sciences & Peking Union Medical College, Tianjin, 300020 China

**Keywords:** Light-chain cardiac amyloidosis, Machine learning, Prognostic model, The revised Mayo 2004 staging system

## Abstract

**Objective:**

To develop and validate a machine learning-based prognostic model that provides enhanced risk stratification for AL cardiac amyloidosis patients beyond existing staging system.

**Methods:**

We conducted a retrospective cohort study of newly diagnosed AL cardiac amyloidosis patients (2006–2024), randomly allocating participants into training and test sets (8:2 ratio). Cardiac involvement required elevated cardiac biomarkers (NT-proBNP > 332 ng/L) or increased wall thickness (mean wall thickness > 12 mm). Using all-cause mortality as the primary endpoint, we compared five machine learning algorithms (support vector machine, CoxBoost, random survival forest, multi-layer perceptron, and k-neighbors classifier) and a traditional Cox model against the 2015 European-modified Mayo staging system.

**Results:**

Among 132 enrolled patients (median age 60 years; 56.8% male), 83 deaths (62.8%) occurred during median 14.5-month follow-up. Feature selection identified six key predictors: cardiac response (36% vs. 15%, *P* < 0.001), complete hematological response (35% vs. 20%, *P* < 0.001), E/e’ ratio (15.0 [12.0, 20.0] vs. 19.0 [15.0, 25.0], *P* = 0.009), left ventricular global longitudinal strain (-14.1 [-11.2 – -16.1] vs. -10.7 [-8.8 – -13.6], *P* = 0.003), serum uric acid (341.0 [292.0–439.0] vs. 429.0 [345.5–518.0], *P* = 0.002), and weight loss (30.6% vs. 65.1%, *P* < 0.001). The CoxBoost model demonstrated superior discrimination (AUC 92%) and calibration (brier score 0.11). Conversely, the predictive value of the revised Mayo 2004 staging system was unsatisfactory, with an AUC of 74% and a brier score of 0.19.

**Conclusions:**

Machine learning incorporating multi-dimensional parameters (including myocardial strain and dynamic clinical variables) provides significantly more accurate prognostication than NT-proBNP/troponin-dependent staging, enabling a new era of personalized risk stratification in AL cardiac amyloidosis.

**Supplementary Information:**

The online version contains supplementary material available at 10.1186/s12911-025-03207-0.

## Introduction

Immunoglobulin light-chain (AL) amyloidosis is a progressive disease characterized by the accumulation of misfolded AL amyloid fibrils in various tissues [1]. AL amyloidosis is a rare type of disease, with an estimated incidence ranging from 5.2 to 13.2 cases per million person-years in the world [2, 3]. Nearly 65 ~ 70% of individuals with AL amyloidosis have cardiac involvement at diagnosis [4, 5]. Patients with AL cardiac amyloidosis display diverse clinical characteristics, ranging from mild lesions to severe restrictive cardiomyopathy [6], and are often susceptible to underdiagnosis. The median survival of advanced AL cardiac amyloidosis patients is < 1 year versus ~ 8 years in AL amyloidosis patients without cardiac involvement [7]. Therefore, the evaluation of the heart, including congestive sign evaluation, cardiac injury biomarkers and imaging, at baseline has been recommended in many clinical guidelines and expert consensuses [1, 8].

Current overall survival prediction for AL cardiac amyloidosis is based on the 2015 European modification of the Mayo 2004 staging system [9]. The revised staging system depends only on cardiac biomarkers. However, AL cardiac amyloidosis is a multisystem disease that may be further exacerbated by additional risk factors [10]. In this study, we aimed to explore the prognostic value of novel echocardiographic and clinical biomarkers, develop machine learning models to predict mortality in AL amyloidosis patients with cardiac involvement, and compare them with the traditional staging system.

## Methods

### Patients

Patients diagnosed with cardiac amyloidosis at Peking Union Medical College Hospital, China, between February 1, 2006, and February 29, 2024, were identified. The diagnosis of AL amyloidosis was confirmed on the basis of 2023 National Comprehensive Cancer Network guidelines [1]. Cardiac involvement was defined on the basis of abnormal cardiac biomarkers and wall thickness [1]. The key exclusion criteria were as follows: (1) patients with other types of amyloidosis, such as transthyretin amyloidosis; (2) patients in whom the diagnosis of AL amyloidosis could not be confirmed due to the unavailability of key diagnostic data, such as a tissue pathology report, mass spectrometry, electron microscopy, or serum free light chain assay results; and (3) patients without cardiac involvement at the time of AL amyloidosis confirmation. This study was approved by the ethics committee of Peking Union Medical College Hospital (approval number: I-22PJ795) and was conducted in accordance with the Declaration of Helsinki. Informed consent was not required because of the retrospective nature of the study.

### Data collection

Clinical data, including demographic characteristics; medical history; general and cardiac symptom evaluation; New York Heart Association functional class assessment; vital sign measurement; laboratory tests covering the kidney, heart, and liver; and electrocardiography examinations, were extracted from medical records. Treatment strategies, responses, and outcomes were also recorded. Weight loss was defined as unintentional loss of at least 5% of the patient’s pre-illness body weight occurring within a 6-month period. The involvement of organs and the hematological and cardiac responses to treatment were assessed according to the 10th International Symposium on Amyloid and Amyloidosis consensus [11] and its updated criteria [12].

### Echocardiography

Image acquisition and parameters are detailed in the Supplementary Methods. All echocardiographic analyses were performed by an experienced reader blinded to the patients’ clinical information. The echocardiographic parameters selected to develop the models were the E/e’ ratio and left ventricular global longitudinal strain (GLS). For the former measure, pulsed-wave Doppler imaging with the sample volume placed between the mitral leaflet tips in the apical 4-chamber view was performed to obtain the peak early diastolic transmitral inflow wave (E) [13]. The peak early diastolic tissue velocity (e’) was obtained via pulsed-wave tissue Doppler imaging of the lateral mitral annulus in the 4-chamber view [13]. Left ventricular GLS were measured via two-dimensional speckle tracking. The GLS value was derived by averaging the peak systolic strain values obtained from the three standard apical views [14].

### Outcomes

Patients were regularly followed up after diagnosis and treatment initiation. The primary outcome of interest was all-cause mortality. The occurrence of outcome events was ascertained by reviewing electronic medical records and/or phone calls to participants. The last follow-up date was April 14, 2024.

### Statistical analysis

Statistical analysis was performed via R Language, version 4.3.2 (R Project for Statistical Computing, Vienna, Austria) and Python, version 3.12. The analyses did not include variables with > 30% missing data, and other missing data were imputed via the random forest algorithm (R package *missForest*). Ultimately, a total of 106 features were included, and the imputation results, which demonstrated a satisfactory imputation effect, are presented in Table S1.

The data were examined for normality via the Shapiro‒Wilk normality test. Continuous characteristics are summarized as the means (standard deviations) when normally distributed or medians (interquartile ranges) when nonnormally distributed. Categorical characteristics were summarized as counts (proportions). Between-group disparities were evaluated with Student’s *t* test for parametric variables and the Mann‒Whitney U test for nonparametric variables. The chi-square test or Fisher’s exact test was used to compare categorical variables as appropriate. All the data were randomly divided into training and test sets at a ratio of 8:2.

### Feature selection

Variables with *P* < 0.1 in the univariable analysis performed on the training set, in addition to clinically established prognostic variables (including New York Heart Association functional class, estimated glomerular filtration rate, bortezomib-based treatment, daratumumab-based treatment, and left ventricular ejection fraction), were selected. These variables were then entered into a least absolute shrinkage and selection operator (LASSO) regression analysis for feature selection (R package *glmnet*). Subsequently, five techniques for analyzing variable importance were performed, including support vector machine (SVM), CoxBoost, random survival forest (RSF), multilayer perceptron (MLP), and k-neighbors classifier (KNN). All the top 10 variables were retained for each analysis method, the results of which were then graphically represented by a Sankey diagram. Correlations between these features were assessed via Spearman’s correlation coefficients and visualized with a heatmap. Owing to the limited sample size in our study and the optimal events per variable required to generate a robust prognostic model, we ultimately identified six feature variables, impacted by both their statistical significance and our existing clinical understanding [15]. The features included in this study are summarized in Table S2.

### Model construction

Five models were built using the machine learning algorithms mentioned above. All algorithms were tenfold cross-validated. Model construction is detailed in the Supplementary Methods. Moreover, a traditional Cox model was built. To evaluate the model’s discriminatory power, we calculated the concordance index (C-index) and the area under the receiver operating characteristic curve (AUC). Other commonly used indices, including sensitivity, specificity, the F1 score, were also calculated. Decision curve analysis (DCA) was performed to confirm the clinical benefits of each model. The calibration accuracy was assessed using calibration curves and the brier score. Five machine learning models and traditional Cox model were compared with the classical prognostic system, the revised Mayo 2004 staging system. The SHapley Additive exPlanations (SHAP) value for survival analysis was used to interpret the machine learning model. The Kaplan‒Meier method was used to analyze survival on the basis of the high–low score groups of the machine learning model, with comparisons performed via the log-rank test. Two-sided *P* values < 0.05 were considered to indicate statistical significance.

## Results

### Patient characteristics

From February 2006 to February 2024, a total of 132 patients diagnosed with AL cardiac amyloidosis at Peking Union Medical College Hospital were enrolled in this study (Figure S1). The median age at diagnosis was 60 years (range: 50.0–65.2 years), and 56.8% of the patients were men. Nearly 70% of patients’ conditions were 2015 European modification of the Mayo 2004 stage IIIa or IIIb. There were 73 (55.3%) and 27 (20.5%) patients with renal and hepatic involvement, respectively. After a median follow-up of 14.5 months (range: 2.6–52.8 months), 83 (62.8%) AL cardiac amyloidosis patients died (Table 1).

**Table 1:** Participant characteristics

	All patients *N* = 132	Survivors *N* = 49	Nonsurvivors *N* = 83	*P* value
Age, y	60.0 (50.0, 65.2)	58.3 (9.2)	57.7 (10.2)	0.753
Male	75 (56.8)	20 (40.8)	55 (66.3)	**0.008**
Diabetes mellitus	11 (8.3)	5 (10.2)	6 (7.2)	0.536
Hypertension	36 (27.3)	15 (30.6)	21 (25.3)	0.646
Current smoking	27 (20.5)	6 (12.2)	21 (25.3)	0.116
**Revised Mayo 2004 stage**				**0.012**
I	7(5.3)	4 (8.2)	3 (3.7)	
II	28 (21.4)	13 (26.5)	15 (18.3)	
IIIa	63 (48.1)	27 (55.1)	36 (43.9)	
IIIb	33 (25.2)	5 (10.2)	28 (34.1)	
**General symptoms**				
Fatigue	37 (28.0)	8 (16.3)	29 (34.9)	**0.036**
Macroglossia	49 (37.1)	13 (26.5)	36 (43.4)	0.08
Periorbital purpura	13 (9.8)	6 (12.2)	7 (8.4)	0.55
Weight loss	69 (52.3)	15 (30.6)	54 (65.1)	**< 0.001**
Paresthesia	31 (23.5)	11 (22.4)	20 (24.1)	0.997
**Cardiac symptoms**				
Chest tightness	61 (46.2)	22 (44.9)	39 (47.0)	0.959
Edema	91 (68.9)	32 (65.3)	59 (71.1)	0.618
Dyspnea	9 (6.8)	7 (14.3)	2 (2.4)	**0.013**
Palpitation	23 (17.4)	12 (24.5)	11 (13.3)	0.159
NYHA class III/IV	44 (33.3)	17 (34.7)	27 (32.5)	0.949
**Organ involvement** **(other than cardiac)**				
Renal	73 (55.3)	27 (55.1)	46 (55.4)	1
Hepatic	27 (20.5)	6 (12.2)	21 (25.3)	0.116
Nervous system	22 (16.7)	5 (10.2)	17 (20.5)	0.197
GI	10 (7.6)	2 (4.1)	8 (9.6)	0.321
Pulmonary	4 (3.0)	2 (4.1)	2 (2.4)	0.627
Soft tissue	58 (43.9)	18 (36.7)	40 (48.2)	0.271
BMI, kg/m^2^	22.0 (20.7, 24.0)	21.7 (2.5)	22.8 (3.1)	**0.028**
Systolic BP, mmHg	102.0 (96.0, 117.2)	108.0 (98.0, 122.0)	100.0 (96.0, 113.0)	0.05
Diastolic BP, mmHg	66.0 (61.0, 75.2)	66.0 (62.0, 78.0)	66.0 (61.0, 73.5)	0.696
**Laboratory examination**				
Hb, g/L	128.0 (113.7, 139.0)	127.0 (107.0, 139.0)	128.0 (118.0, 138.5)	0.603
WBC, ×10^6^/L	6.2 (4.7, 7.5)	6.3 (5.1, 7.4)	6.1 (4.4, 7.6)	0.972
eGFR, mL/min	69.9 (54.3, 83.5)	72.2 (57.7, 85.1)	66.2 (53.4, 82.9)	0.22
Urine albumin, g/24 h	1.1 (0.2, 2.7)	1.3 (0.2, 3.3)	0.9 (0.3, 2.7)	0.827
Creatinine, µmol/L	76.0 (63.7, 99.5)	69.0 (60.0, 83.0)	82.0 (67.5, 108.5)	**0.008**
UA, µmol/L	395.5 (313.0, 488.0)	341.0 (292.0, 439.0)	429.0 (345.5, 518.0)	**0.002**
K^+^, mmol/L	4.0 (3.8, 4.3)	4.0 (3.7, 4.2)	4.1 (3.8, 4.4)	**0.032**
Ca^2+^, mmol/L	2.2 (2.1, 2.3)	2.2 (2.2, 2.3)	2.2 (2.1, 2.3)	0.077
ALP, U/L	92.5 (71.7, 142.0)	86.0 (66.0, 111.0)	108.0 (76.0, 152.0)	**0.031**
Total bilirubin, µmol/L	14.6 (8.8, 19.7)	12.2 (7.4, 17.9)	15.3 (10.6, 22.8)	0.111
Direct bilirubin, µmol/L	5.0 (2.9, 7.9)	4.5 (2.1, 5.6)	5.8 (3.4, 9.4)	**0.007**
Serum albumin, g/L	36.0 (31.0, 40.2)	36.0 (30.0, 41.0)	36.0 (32.0, 40.0)	0.858
Albumin/globulin ratio	1.5 (0.54)	1.5 (0.5)	1.5 (0.5)	0.948
LD, U/L	211.5 (183.7, 257.7)	229.0 (188.0, 280.0)	210.0 (178.0, 240.0)	0.093
Serum free κ chain, mg/L	20.2 (11.1, 129.0)	16.9 (9.8, 41.5)	36.8 (12.0, 175.0)	**0.045**
Serum free λ chain, mg/L	247.1 (65.8, 436.2)	188.5 (37.8, 327.5)	282.6 (137.3, 470.3)	0.117
dFLC, mg/L	251.8 (118.1, 506.1)	179.3 (62.8, 425.5)	274.9 (151.5, 514.0)	**0.039**
Serum free κ/λ ratio	0.2 (0.0, 7.3)	0.1 (0.0, 1.1)	0.2 (0.0, 9.9)	0.091
Troponin I, µg/L	0.1 (0.0, 0.5)	0.1 (0.0, 1.2)	0.1 (0.0, 0.3)	0.944
NT-proBNP, pg/mL	4378.0 (1773.7, 8583.7)	2994.0 (1425.0, 6108.0)	5284.0 (2665.5, 11781.0)	**0.001**
**Electrocardiography**				
Low voltage	57 (43.2)	17 (34.7)	40 (48.2)	0.183
Atrial fibrillation	21 (15.9)	11 (22.4)	10 (12.0)	0.183
Pseudoinfarction	18 (13.6)	7 (14.3)	11 (13.3)	1
First-degree AV block	4 (3.0)	2 (4.1)	2 (2.4)	0.627
Second-degree AV block	3 (2.3)	0 (0.0)	3 (3.6)	0.294
Third-degree AV block	1 (0.8)	1 (2.0)	0 (0.0)	0.371
LBBB	9 (6.8)	3 (6.1)	6 (7.2)	1
RBBB	8 (6.1)	5 (10.2)	3 (3.6)	0.147
**Echocardiography**				
LA mass, mL	50.4 (38.2, 64.5)	48.9 (39.0, 57.0)	51.5 (37.6, 69.3)	0.156
LA mass index, mL/m^2^	30.4 (22.6, 38.6)	30.2 (22.6, 35.3)	31.3 (22.8, 39.5)	0.193
RATD, cm	3.7 (0.8)	3.4 (0.7)	3.8 (0.8)	**0.005**
RA mass, mL	42.9 (27.6, 54.9)	36.8 (18.2)	46.6 (21.8)	**0.009**
LVEDd, mm	42.0 (38.0, 45.0)	42.0 (39.0, 46.0)	41.0 (38.0, 44.5)	0.286
LVLD, mm	73.2 (7.7)	73.0 (69.7, 76.0)	75.0 (68.0, 79.0)	0.206
RVEDd, mm	30.7 (7.1)	29.9 (6.8)	31.2 (7.3)	0.319
RVLD, mm	61.6 (9.9)	60.3 (9.3)	62.4 (10.1)	0.232
IVSd, mm	13.9 (3.2)	13.7 (3.3)	14.0 (3.2)	0.543
PWTd, mm	13.0 (11.0, 15.0)	13.0 (11.0, 15.0)	13.0 (11.0, 15.0)	0.283
RVFWT, mm	6.0 (5.0, 7.0)	6.0 (5.0, 7.0)	6.0 (5.0, 7.0)	0.775
LV EF, %	54.1 (11.9)	56.9 (11.4)	52.4 (11.9)	**0.033**
TAPSE, cm	14.1 (4.7)	15.3 (4.5)	13.4 (4.7)	**0.024**
E/A ratio	2.0 (1.0, 2.2)	1.4 (1.0, 2.0)	2.0 (1.1, 2.4)	**0.015**
E/e’ ratio	18.0 (13.8, 24.5)	15.0 (12.0, 20.0)	19.0 (15.0, 25.0)	**0.009**
TRV, m/s	2.5 (2.2, 2.8)	2.5 (2.2, 2.9)	2.6 (2.2, 2.7)	0.834
LV GLS, %	-11.6 (-9.1, -15.2)	-14.1 (-11.2, -16.1)	-10.7 (-8.8, -13.6)	**0.003**
LV GCS, %	-17.9 (-7.5)	-18.8 (-6.6)	-17.3 (-8.0)	0.252
LV GRS, %	11.6 (6.9, 16.3)	12.9 (8.1, 21.7)	10.2 (5.7, 14.9)	**0.013**
Pericardial effusion	91 (68.9)	35 (71.4)	56 (67.5)	0.779
sPAP, mmHg	32.7 (26.6, 39.2)	32.5 (28.1, 40.0)	32.8 (24.6, 38.3)	0.765
Granular sparking myocardial appearance	88 (66.6)	40 (81.6)	48 (57.8)	**0.009**
**Hematological medication**				
ASCT	3 (2.3)	2 (4.1)	1 (1.2)	0.555
Bortezomib based	79 (59.8)	33 (67.3)	46 (55.4)	0.243
Daratumumab based	16 (12.1)	10 (20.4)	6 (7.2)	**0.049**
IMiDs based	16 (12.1)	4 (8.2)	12 (14.5)	0.427
Others	19 (14.4)	9 (18.4)	10 (12.0)	0.458
**Cardiac medication**				
ACEI/ARB	3 (2.3)	1 (2.0)	2 (2.4)	1
Beta-blocker	18 (13.6)	5 (10.2)	13 (15.7)	0.535
CCB	6 (4.5)	5 (10.2)	1 (1.2)	**0.026**
Diuretic	110 (83.3)	36 (73.5)	74 (89.2)	**0.036**
**Other medication**				
Allopurinol	7 (5.3)	3 (6.1)	4 (4.8)	0.710
**Hematologic response**				
CR				
1 month	18 (13.6)	11 (22.4)	7 (8.4)	**0.045**
3 months	30 (22.7)	17 (34.7)	13 (15.7)	**0.021**
6 months	43 (32.6)	28 (57.1)	15 (18.1)	**< 0.001**
Best response	55 (41.7)	35 (71.4)	20 (24.1)	**< 0.001**
VGPR				
1 month	1 (0.8)	1 (2.0)	0 (0.0)	0.371
3 months	2 (1.5)	1 (2.0)	1 (1.2)	1
6 months	6 (4.5)	5 (10.2)	1 (1.2)	**0.026**
Best response	12 (9.1)	7 (14.3)	5 (6.0)	0.127
PR				
1 month	20 (15.2)	8 (16.3)	12 (14.5)	0.97
3 months	20 (15.2)	11 (22.4)	9 (10.8)	0.122
6 months	10 (7.6)	2 (4.1)	8 (9.6)	0.321
Best response	12 (9.1)	2 (4.1)	10 (12.0)	0.209
No				
1 month	19 (14.4)	9 (18.4)	10 (12.0)	0.458
3 months	12 (9.1)	6 (12.2)	6 (7.2)	0.36
6 months	8 (6.1)	3 (6.1)	5 (6.0)	1
**Cardiac response**				
Remission				
1 month	13 (9.8)	10 (20.4)	3 (3.6)	**0.004**
3 months	22 (16.7)	17 (34.7)	5 (6.0)	**< 0.001**
6 months	33 (25.0)	26 (53.1)	7 (8.4)	**< 0.001**
Best response	51 (38.6)	36 (73.5)	15 (18.1)	**< 0.001**
Progression				
1 month	34 (25.8)	14 (28.6)	20 (24.1)	0.717
3 months	29 (22.0)	10 (20.4)	19 (22.9)	0.908
6 months	26 (19.7)	5 (10.2)	21 (25.3)	0.06

ALP, alkaline phosphatase; AV, atrioventricular; ASCT, autologous stem cell transplantation; ACEI/ARB, angiotensin-converting enzyme inhibitor/angiotensin II receptor blocker; BMI, body mass index; BP, blood pressure; CCB, calcium channel blocker; CR, complete remission; dFLC, difference in free light chains; eGFR, estimated glomerular filtration rate; EF, ejection fraction; GI, gastrointestinal; GLS, global longitudinal strain; GCS, global circumferential strain; GRS, global radial strain; Hb, hemoglobin; IMiDs, immunomodulators; LA, left atrium; LBBB, left bundle branch block; LD, lactic acid dehydrogenase; LV, left ventricle; LVEDd, left ventricular end-diastolic diameter; LVLD, left ventricular long diameter; NYHA, New York Heart Association; NT-proBNP, N-terminal pro-brain natriuretic peptide; RA, right atrium; RATD, right atrium diameter; RBBB, right bundle branch block; RVEDd, right ventricular end-diastolic diameter; RVLD, left ventricular long diameter; RVFWT, right ventricular free wall thickness; IVSd, interventricular septum in diastole; PWTd, posterior wall thickness in diastole; PR, partial remission; TAPSE, tricuspid annular plane systolic excursion; TRV, tricuspid regurgitation velocity; UA, serum uric acid; VGPR, very good partial remission; WBC, white blood cell; sPAP, systolic pulmonary artery pressure

Compared with surviving patients, nonsurviving patients were more likely to be male (40.8% vs. 66.3%, *P* = 0.008); to experience weight loss (30.6% vs. 65.1%, *P* < 0.001); to have higher levels of serum creatinine [69.0 (60.0–83.0) vs. 82.0 (67.5–108.5), *P* = 0.008], serum UA [341.0 (292.0–439.0) vs. 429.0 (345.5–518.0), *P* = 0.002], alkaline phosphatase [86.0 (66.0–111.0) vs. 108.0 (76.0–152.0), *P* = 0.031], difference in free light chains [179.3 (62.8–425.5) vs. 274.9 (151.5–514.0), *P* = 0.039], and N-terminal pro-brain natriuretic peptide (NT-proBNP) [2994.0 (1425.0–6108.0) vs. 5284.0 (2665.5–11781.0), *P* = 0.001]; and to exhibit worse ventricular systolic and diastolic function (*P* < 0.05). Interestingly, nonsurviving patients had a less granular sparkling myocardial appearance (81.6% vs. 57.8%, *P* = 0.009) on echocardiography. Additionally, nonsurviving patients were less likely to receive daratumumab-based treatment (20.4% vs. 7.2%, *P* = 0.049) and achieve hematologic complete response and cardiac response than survivors were (*P* < 0.05). The training set and test set did not differ significantly (*P* > 0.05), and a partial feature comparison is shown in Table S3.

### Feature selection

**Fig. 1:**
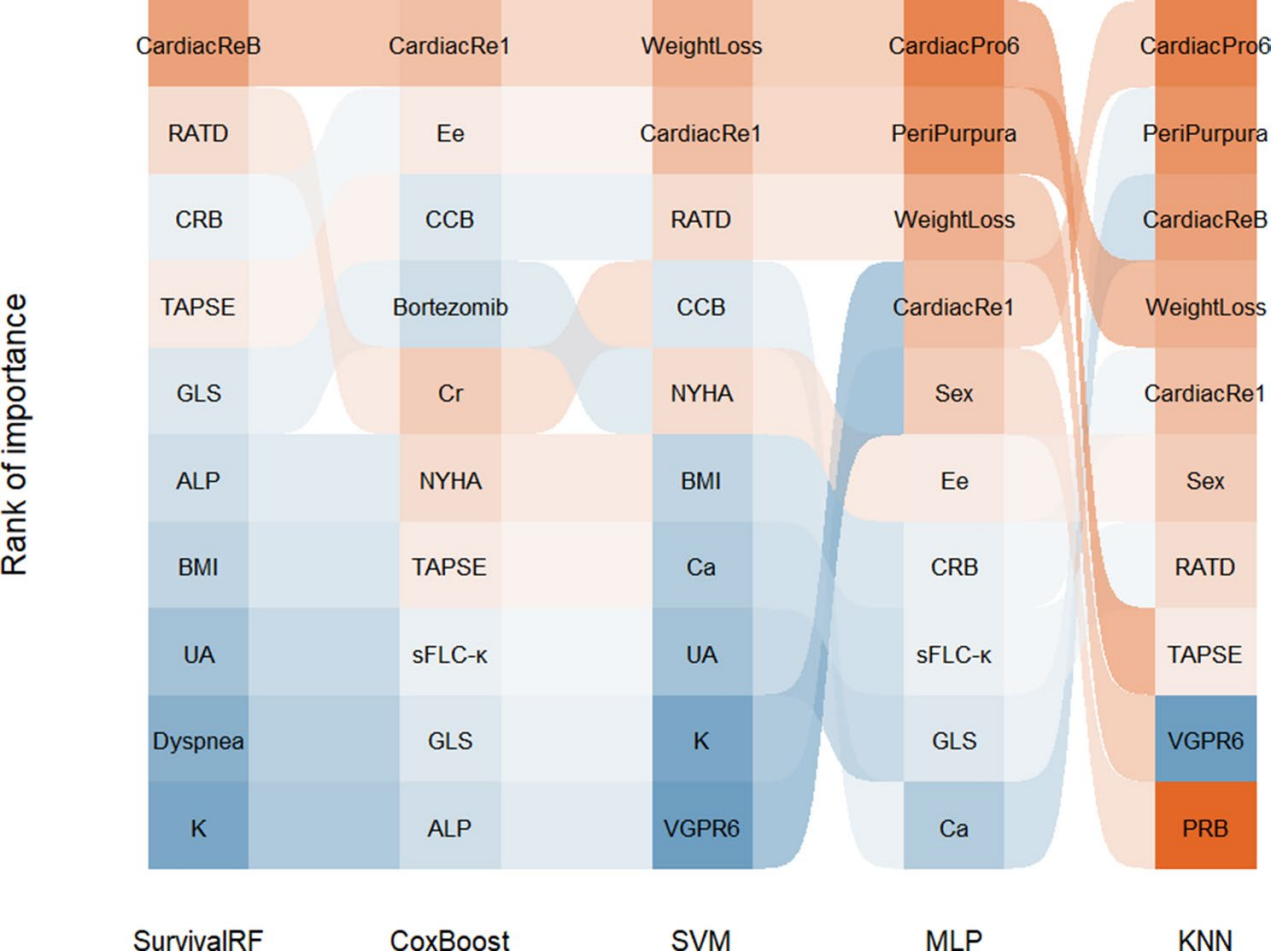
Sankey diagram for the top 10 variables from the five variable importance ranking methods. Bortezomib, Bortezomib-based treatment; CardiacRe1, cardiac remission (1 month); CardiacReB, cardiac response; CardiacPro6, cardiac progression (6 months); CRB, complete hematologic remission; Ee, E/e’ ratio; KNN, k-neighbors classifier; MLP, multilayer perceptron; PeriPurpura, periorbital purpura; sFLC-κ, serum free κ chain; SVM, support vector machine; SurvivalRF, random survival forest. The other abbreviations are the same as those in Table 1

Thirty-seven features in univariate analysis were initially selected as potential predictors (Table S2). Further feature screening was performed using LASSO analysis (Figure S2). This process yielded 28 features with nonzero coefficients. Using five variable importance analysis methods, we selected the top 10 variables from each method (Fig. 1). Considering statistical significance and clinical knowledge, six variables were ultimately selected, including weight loss, serum UA levels, the E/e’ ratio, left ventricular GLS, best hematologic response, and best cardiac response. Spearman’s correlation coefficients were calculated (Figure S3).

### Model construction

We constructed five machine learning models with tenfold cross-validation and a traditional Cox model. The performance of these predictive models is summarized in Table 2. In the training set, compared with the other models, MLP demonstrated relatively better performance, with an AUC of 99%, followed by random survival forest, with an AUC of 89%. The predictive value of the traditional model, the 2015 European modification of the Mayo 2004 staging system, was unsatisfactory, with an AUC of 60% (Figure S4).

**Table 2:** Prediction performances of the different models

	Cox	SVM	CoxBoost	MLP	RSF	KNN	Revised Mayo 2004 stage
**Training set**							
AUC	0.86	0.87	0.86	0.99	0.89	0.75	0.60
Brier score	0.36	0.15	0.16	0.14	0.17	0.18	0.25
C-index	0.79	0.78	0.79	0.73	0.81	0.63	0.56
SE	0.70	0.70	0.70	0.77	0.73	0.53	0.31
SP	0.84	0.84	0.84	0.97	0.89	0.82	0.87
F1 score	0.78	0.78	0.78	0.86	0.81	0.65	0.45
**Test set**							
AUC	0.92	0.94	0.92	0.89	0.9	0.66	0.74
Brier score	0.37	0.10	0.11	0.18	0.11	0.23	0.19
C-index	0.72	0.73	0.72	0.66	0.69	0.60	0.64
SE	0.75	0.75	0.75	0.68	0.75	0.56	0.46
SP	0.90	0.90	0.90	0.80	0.90	0.60	1.00
F1 score	0.80	0.82	0.82	0.75	0.82	0.62	0.63

AUC, area under the receiver operating characteristic curve; C-index, concordance index; Cox, Cox proportional-hazards regression; MLP, multilayer perceptron; SE, sensitivity; SP, specificity; KNN, k-neighbors classifier; RSF, random survival forest; SVM, support vector machine

**Fig. 2:**
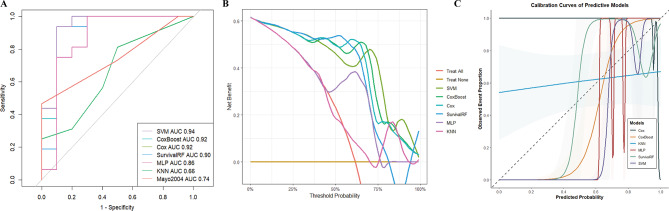
Classified multimodel comprehensive analysis in the test set. (**A**) ROC curve for five machine learning models, traditional Cox model, and the European 2015 modification of the Mayo 2004 staging system. (**B**) DCA for five machine learning models and traditional Cox model. (**C**) Calibration curve for five machine learning models and traditional Cox model. The abbreviations are the same as those in Table 2

**Fig. 3:**
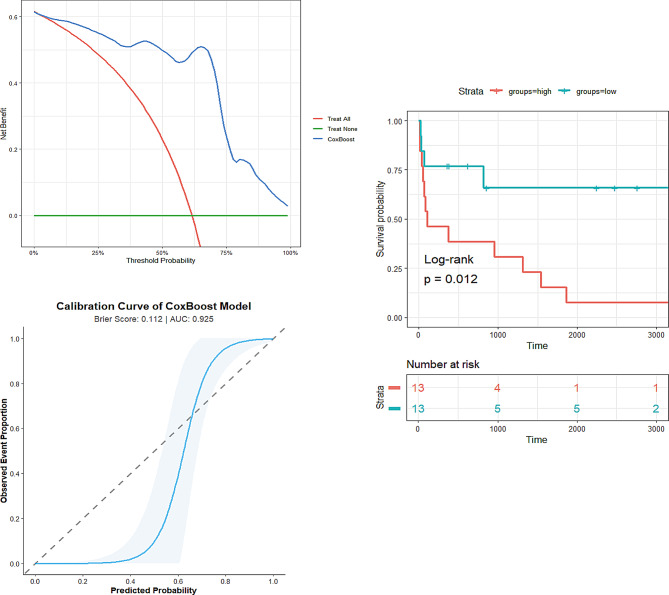
CoxBoost model performance in the test set. (**A**) DCA curves. (**B**) Calibration curve. (**C**) Kaplan‒Meier survival curves of patients stratified into risk groups. Differences in overall survival between the high- and low-risk groups were evaluated by the log-rank test. AUC, area under the receiver operating characteristic curve; DCA, decision curve analysis; ROC, receiver operating characteristic curve

In the test set, the SVM model demonstrated superior discriminative ability compared to other models, achieving an AUC of 94%, followed by CoxBoost and traditional Cox model (AUC: 92%). However, both the SVM and traditional Cox models exhibited suboptimal calibration in the calibration curves (Fig. 2). Notably, CoxBoost showed excellent calibration, with a brier score of 0.11 (Fig. 3). In contrast, the revised Mayo 2004 staging system demonstrated significantly inferior predictive performance, with modest discrimination (AUC = 74.6%) and poor calibration (brier score = 0.19) (Table 2).

**Fig. 4:**
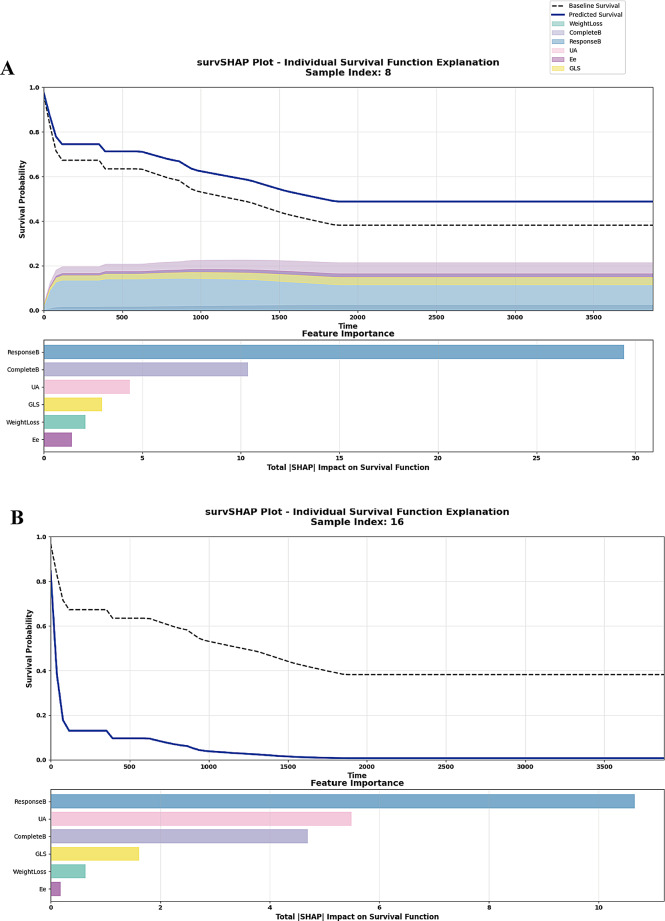
SHAP analysis of the CoxBoost model. SHAP-based risk prediction and feature importance ranking for a low risk (**A**) and a high risk patient (**B**). SHAP, Shapley additive explanations. The abbreviations are the same as those in Table 1

The CoxBoost model was selected as the representative machine learning model in this study. In the test set, CoxBoost model shows consistently outperforming reference thresholds across all decision points in the DCA curve, while its calibration curve revealed excellent agreement between predicted and observed probabilities (Fig. 3). The Kaplan‒Meier curve effectively distinguished between the high- and low-risk groups (log-rank *P* = 0.012) (Fig. 3). To further interpret the machine learning-based model, we employed two real-world examples to demonstrate the interpretability of our model, showcasing the dynamic, time-dependent contribution of each variable to the final prediction (Fig. 4).

## Discussion

Our study yielded three key findings: (1) Six routinely evaluated clinical parameters were identified as significant predictors of overall survival in AL cardiac amyloidosis; (2) Among various machine learning approaches, the CoxBoost algorithm showed particularly strong prognostic performance; (3) The CoxBoost-based model outperformed the conventional Mayo 2004 staging system in prognostic accuracy.

AL amyloidosis is a life-threatening disease, and its prognosis is dependent on the presence and severity of heart involvement. Currently, the therapy goal of AL cardiac amyloidosis is to suppress further production of amyloidogenic light chains, allowing organ recovery and improving overall survival. The hematologic response is fundamental to the cardiac response, both of which have been shown to improve the prognosis of AL cardiac amyloidosis. Efstathios Kastritis et al. enrolled 227 consecutive AL amyloidosis patients and reported that those who experienced rapid and deep hematologic responses generally experienced organ responses as well [16]. In a study that included 67 patients diagnosed with advanced light chain cardiac amyloidosis via the Mayo 2004 staging system, Grzegorz Charlinski et al. reported that patients achieving a very good partial response, a complete hematological remission, or a cardiac remission showed significantly longer overall survival [17]. In our cohort, compared with the nonsurvival group, the survival group had a greater proportion of patients with complete hematologic remission and cardiac remission (*P* < 0.05). We performed five variable importance ranking techniques, all of which consistently retained cardiac remission (assessed at 1 month and/or as best response) as an important variable, underscoring its prognostic significance in in AL cardiac amyloidosis patients after treatment. Beyond cardiac remission, complete hematologic remission was identified as the second most important treatment response variable by the RSF algorithm—a method specifically designed for survival analysis. Therefore, cardiac response and complete hematologic response were identified as crucial prognostic indicators. The New York Heart Association functional class was excluded due to its strong correlation with the cardiac response, defined by dynamic changes in one of the following indicators: New York Heart Association functional class, NT-proBNP levels, or ejection fraction. Daratumumab-based treatment have not retained in ranking analysis, while bortezomib-based treatment and serum free κ chain was excluded for due to its strong correlation with the hematologic response.

Next, we included E/e’ and GLS as prognostic indicators. In the heart, light chain amyloid fibril deposition expands the extracellular space, produces cytotoxic effects, and leads to changes in cardiac structure and function [18]. The classical echocardiography of AL cardiac amyloidosis includes thickening of the left ventricle, reduced diastolic function, a granular sparkling myocardial appearance, enlarged atria, and reduced systolic function [8]. In our cohort, compared with the survival group, the nonsurvival group had worse diastolic (E/e’ ratio, E/A ratio) and systolic (left ventricular ejection fraction, GLS, tricuspid annular plane systolic excursion) functions (*P* < 0.05). Following further screening and variable importance ranking analysis, the E/e’ ratio, GLS, and tricuspid annular plane systolic excursion were retained. Several studies have demonstrated the prognostic value of the E/e’ ratio in AL amyloidosis. Changhui Lei et al. analyzed 74 AL amyloidosis patients and reported that the E/e’ ratio was an independent predictor of survival after adjusting for cardiac troponin T and GLS [19]. Previously, they researched 55 AL amyloidosis patients with cardiac involvement and concluded that the E/e’ ratio in the nonsurvival group was significantly greater than that in the survival group and was identified as a significant predictor of mortality via multivariate Cox analysis [20]. The prognostic value of GLS in AL amyloidosis have been reported in several studies. In an analysis of 94 patients with advanced AL amyloidosis, Katherine Lee Chuy et al. found that GLS serves as an independent predictor of overall survival, providing incremental value beyond the Mayo 2012 Staging system [21]. Similarly, Xinhao Li et al. conducted a study involving 140 AL amyloidosis patients with cardiac involvement and concluded that GLS was independently associated with major adverse cardiac events [22]. In contrast, the prognostic value of TAPSE in AL amyloidosis remains unclear [23]. Therefore, based on the established prognostic value of E/e’ and GLS—and considering the uncertain significance of TAPSE in this population—the E/e’ ratio and GLS were selected for inclusion in the final model.

AL cardiac amyloidosis exhibits multi-organ involvement, most commonly affecting the heart, kidneys, liver, and digestive system. In AL amyloidosis patients, extra amyloid fibrils can deposit in the tissue interstitium, which causes functional cell atrophy and then organ dysfunction. Serum UA level, a biomarker reflecting cardiac and renal dysfunction, has been identified as an independent prognostic factor in AL amyloidosis patients [24]. Shaji Kumar et al. studied 1977 patients with AL amyloidosis at the Mayo Clinic between April 1960 and August 2006 and 293 patients who underwent peripheral blood stem cell transplant for systemic AL amyloidosis to examine the prognostic value of serum UA levels. Those authors reported that patients with UA levels greater than 8 mg/dL had shorter survival than the remaining patients did (*P* < 0.001), and UA levels had independent prognostic value after adjusting for troponin T and NT-proBNP levels [24]. Furthermore, they developed a model incorporating troponin T, NT-proBNP, and UA levels to identify AL amyloidosis patients at risk of early mortality in the training cohort of 459 participants and then confirmed it in a validation cohort of 313 participants [25]. Another multisystem indicator that often arises is weight loss, which can be a combined sign of gastrointestinal tract involvement, cardiac cachexia, and/or liver infiltration [26]. In a study conducted by Suzanne R Hayman et al. at the Mayo Clinic, patients with AL amyloidosis and malabsorption syndrome were analyzed carefully, and weight loss was identified as a key indicator of poor survival in these individuals [27]. In our cohort, the proportion of weight loss was greater in the nonsurvival group than in the survival group (30.6% vs. 65.1%, *P* < 0.001). The estimated glomerular filtration rate were removed from the importance ranking analysis, whereas shortness of breath were removed because of their nonspecificity. Creatinine and periorbital purpura were also excluded based on limited evidence regarding their prognostic value. Finally, we integrated serum UA levels and weight loss into our study.

We developed five machine learning models and traditional Cox model for predicting patient prognosis. The CoxBoost model achieved the highest AUC (92%) in the test set, indicating strong performance in distinguishing between surviving and non-surviving patients. CoxBoost model’s consistent performance in DCA curves, indicate a comparable precision-recall balance and robust clinical utility across diverse healthcare settings. The calibration curve demonstrates excellent agreement between the predicted and observed event probabilities for the CoxBoost model, which is further supported by its strong brier score of 0.11. In contrast, the 2015 European modification of the Mayo 2004 staging system**—**based on NT-proBNP and troponin I levels**—**showed relatively poor performance, with an AUC of 74%, an F1 score of 0.63, and a brier score of 0.19. The performance of developed machine learning model, especially the CoxBoost model, was superior to that of the traditional Mayo system.

Our research also has several limitations. Given the rarity of AL cardiac amyloidosis, this study was necessarily limited by a small sample size. To mitigate this limitation, we systematically screened and enrolled cases spanning an 18-year period. Furthermore, comprehensive clinical data were collected to enhance the dataset’s robustness. Future multicenter prospective studies with larger cohorts will be required. Second, the therapeutic regimens varied across different years [28, 29], which may have influenced the outcomes. Specifically, the availability of novel agents such as bortezomib and daratumumab for AL amyloidosis treatment changed over time in our clinical setting. However, it’s important to note that treatment characteristics were not incorporated as variables in our final predictive model, but were rather presented as descriptive features of the study population. Third, important variables, including cardiac troponin T and brain natriuretic peptide, were excluded from this study due to unacceptably high rates of missing data in our cohort. Consequently, neither the Mayo 2012 staging system (which incorporates cardiac troponin T) [30] nor the Boston 2019 staging system (which includes brain natriuretic peptide) [31] could be applied, potentially introducing bias into our analysis. However, we employed the 2015 European-modified Mayo 2004 staging system**—**a recommended model for AL cardiac amyloidosis**—**for severity assessment [29]. Fourth, cardiac magnetic resonance (CMR) indicators**—**particularly advanced imaging techniques such as myocardial T1, T2, and extracellular volume mapping**—**were excluded due to a high rate of missing data (only 66 patients underwent CMR). CMR enables a more comprehensive evaluation of myocardial tissue characteristics in patients with AL cardiac amyloidosis and may provide valuable prognostic insights. Future studies integrating these advanced imaging techniques are strongly recommended. Fifth, although we collected detailed clinical data, follow-up echocardiographic data were not available for most patients. Future studies can prioritize the collection of longitudinal echocardiographic data to better evaluate cardiac function and its relationship with adverse outcomes in AL amyloidosis patients.

## Conclusions

While NT-proBNP and troponin remain cornerstone biomarkers, our study demonstrates how machine learning can extract additional prognostic value from complex clinical data. The high-performance CoxBoost model complements and enhances the revised Mayo staging system, marking a significant advance in risk assessment precision.

## Supplementary Information

Below is the link to the electronic supplementary material.

Supplementary Material 1

Supplementary Material 2

Supplementary Material 3

Supplementary Material 4

Supplementary Material 5

## Data Availability

The data supporting this study’s findings are available on request from the corresponding author [Wei Chen]. The data are not publicly available because they contain information that could compromise the privacy of research participants.

## Abbreviations

AL amyloidosis: Immunoglobulin light-chain amyloidosis
AUC: The area under the receiver operating characteristic curve
CMR: Cardiac magnetic resonance
C-index: The concordance index
DCA: Decision curve analysis
GLS: Global longitudinal strain
KNN: K-neighbors classifier
LASSO: Least absolute shrinkage and selection operator
NT-proBNP: N-terminal pro-brain natriuretic peptide
MLP: Multilayer perceptron
RSF: Random survival forest
SVM: Support vector machine
SHAP: The SHapley Additive exPlanations
