# Supplementary material for: Beyond NT-proBNP and troponin: How machine learning redefines light-chain cardiac amyloidosis risk assessment
Source: BMC Med Inform Decis Mak. 2025 Oct 9;25:367. doi: 10.1186/s12911-025-03207-0 (PMC12512860; doi:10.1186/s12911-025-03207-0)
Supplement: Supplementary file 1 — Supplementary Material 1 [file 12911_2025_3207_MOESM1_ESM.docx]

**SUPPLEMENTAL METHODS**

**Echocardiography**

All participants underwent echocardiography on a Vivid E9 ultrasound machine (GE Medical Systems, Milwaukee, Wisconsin) at baseline. The image quality was optimized by adjusting the temporal resolution (50 to 70 frames/s) with a 3.5 MHz transductor. Three consecutive heart cycles were recorded. Atrial and ventricular structure and ventricular systolic, diastolic, and strain were measured according to published recommendations according to published recommendations[1, 2]. Myocardial texture was observed via standard two-dimensional echocardiographic imaging, without the application of tissue harmonics[3]. A positive finding was defined as a increased, hyper-refractile, and fine speckled echogenicity of the myocardium, distinct from the normal myocardial texture[4].

**Model construction**

SVM was processed with the *e1071* R package (version 1.7.14) and class (version 7.3.22). *tune.svm* was adopted to optimize the settings parameter with the cost between 0.001 and 10 and the kernel of “linear”. The SVM was developed on the basis of the best cost.

CoxBoost was processed with the *CoxBoost* (version 1.5) R package. *cv.CoxBoost* was used to optimize the penalty and step parameters. On the basis of the optimized parameters, *CoxBoost* was applied to the training set.

RSF was completed with the R package *randomForestSRC* (version 3.2.3). The *tune* function was adopted to optimize the tunesize parameter. RSF was developed with the optimal nodesize and mtry parameters.

The MLP was developed with *h2o* (version 3.44.0.3) in the Java virtual environment. First, *h2o.init* was used to initiate *h2o* and adjust the model’s memory setting to 8G. *as.h2o* was used to transform the data format to *the h2o* data type. Next, *h20.deeplearning* was used to tune the parameters and develop the model with the rectifier activation function, with an iteration time of 10 and a hidden layer size of c(20,20,20).

The KNN algorithm was processed with *e1071* (version 1.7.14) and class (version 7.3.22). On the basis of the “knn” method and tenfold cross-validation, the *train* function was adopted for the KNN model.

**References**

1. Mitchell C, Rahko PS, Blauwet LA, Canaday B, Finstuen JA, Foster MC, Horton K, Ogunyankin KO, Palma RA, Velazquez EJ: Guidelines for Performing a Comprehensive Transthoracic Echocardiographic Examination in Adults: Recommendations from the American Society of Echocardiography. J Am Soc Echocardiogr 2019, 32(1):1-64.

2. Liu JE, Barac A, Thavendiranathan P, Scherrer-Crosbie M: Strain Imaging in Cardio-Oncology. JACC CardioOncol 2020, 2(5):677-689.

3. Selvanayagam JB, Hawkins PN, Paul B, Myerson SG, Neubauer S: Evaluation and management of the cardiac amyloidosis. J Am Coll Cardiol 2007, 50(22):2101-2110.

4. Dorbala S, Ando Y, Bokhari S, Dispenzieri A, Falk RH, Ferrari VA, Fontana M, Gheysens O, Gillmore JD, Glaudemans AWJM et al: ASNC/AHA/ASE/EANM/HFSA/ISA/SCMR/SNMMI Expert Consensus Recommendations for Multimodality Imaging in Cardiac Amyloidosis: Part 1 of 2-Evidence Base and Standardized Methods of Imaging. Circ Cardiovasc Imaging. 2021 Jul;14(7):e000029.

Figure S1. Flowchart of the selection process and modeling strategy. PUMCH, Peking Union Medical College Hospital; SHAP, Shapley additive explanation.

Figure S2. LASSO regression was used to select the representative features. (A) LASSO coefficient profiles for 37 variables. (B) Selection of the tuning parameter (λ) in the LASSO model with tenfold cross-validation. We selected the log(λ) where the variables correspond to the dashed line on the right. LASSO, the least absolute shrinkage and selection operator.

Figure S3. Correlation heatmaps between the top 10 variables from the five variable importance ranking methods. Features with strong correlations are shown in dark orange (positive correlation) or dark blue (negative correlation). Features with cross had nonsignificant *P*-values in Spearman’s correlation analysis. The abbreviations are the same as those in Table 1 and Figure 1.

Figure S4. Classified multimodel comprehensive analysis in the training set. (A) ROC curve for five machine learning models, traditional Cox model and the European 2015 modification of the Mayo 2004 staging system, (B) DCA for five machine learning models and traditional Cox model, and (C) calibration curve for five machine learning models and traditional Cox model. The abbreviations are the same as those in Table 1 and Figure 2.

Table S1. Results of data imputation

| *MissForest* imputation results  (OBB error) | NRMSE | PFC |
| --- | --- | --- |
|  | 0.615 | 0.087 |

Abbreviations: OBB error, out of box error; NRMSE, normalized root-mean-square error; PFC, proportion of falsely classified entries.

Table S2. Enrolled features in this study

|  | Feature name |
| --- | --- |
| Features with missing value ≤30%  (106 features) | Age, sex, diabetes mellitus, hypertension, smoking, revised Mayo 2004 stage, general symptoms (fatigue, macroglossia, periorbital purpura, weight loss, paresthesia), cardiac symptoms (chest tightness, edema, short of breath, palpitation), NYHA class, organ involvement (renal, hepatic, nervous system, GI, soft tissue), BMI, systolic BP, diastolic BP, laboratory examination (Hb, WBC, eGFR, urine albumin, creatinine, UA, K^+^, Ca^2+^, ALP, total bilirubin, direct bilirubin, serum albumin, albumin/globulin ratio, LD, serum free κ chain, serum free λ chain, dFLC, serum free κ/λ ratio, troponin I, NT-proBNP), electrocardiography (low voltage, atrial fibrillation, pseudoinfarction, first-degree AV block, second-degree AV block, third-degree AV block, LBBB, RBBB), echocardiography (LA mass, LA mass index, RATD, RA mass, LVEDd, LVLV, RVEDd, RVLD, IVSd, PWTd, RVFWT, LV EF, TAPSE, E/A ratio, E/e’ ratio, TRV, LV GLS, LV GCS, LV GRS, Pericardial effusion, sPAP, granular sparking myocardial appearance), hematological medication (ASCT, Bortezomib based, Daratumumab based, IMiDs based, others), cardiac medication (ACEI/ARB, Beta-blocker, CCB, diuretic, hematologic response at 1 month, 3 months, 6 months (CR, VGPR, PR, No), cardiac response at 1 month, 3 months, 6 months (remission, progression), the best hematologic response (CR, VGPR, PR), the best cardiac response (remission) |
| Candidate variables for LASSO  (37 features) | Sex, fatigue, periorbital purpura, weight loss, short of breath, NYHA class, BMI, SBP, eGFR, creatinine, UA, K^+^, Ca^2+^, ALP, serum free κ chain, dFLC, NT-proBNP, RATD, RA mass, LV EF, TAPSE, E/e’ ratio, LV GLS, granular sparking myocardial appearance, bortezomib based treatment, daratumumab based treatment, CCB, diuretic, hematologic CR (6 months and the best), cardiac remission (1 month, 3 months, 6 months and the best), cardiac progression (6 months), hematologic VGPR (6 months), hematologic PR (the best) |
| LASSO analysis  (28 features) | Sex, fatigue, periorbital purpura, weight loss, short of breath, NYHA class, BMI, SBP, creatinine, UA, K^+^, Ca^2+^, ALP, serum free κ chain, RATD, TAPSE, E/e’ ratio, LV GLS, granular sparking myocardial appearance, bortezomib based treatment, daratumumab based treatment, CCB, hematologic CR (the best), cardiac remission (1 month, the best), cardiac progression (6 months), hematologic VGPR (6 months), hematologic PR (the best) |
| Top 10 variables in six feature ranking methods  (24 features) | Sex, periorbital purpura, weight loss, short of breath, NYHA class, BMI, creatinine, UA, K^+^, Ca^2+^, ALP, serum free κ chain, RATD, TAPSE, E/e’ ratio, LV GLS, bortezomib based treatment, CCB, hematologic CR (the best), cardiac remission (1 month, the best), cardiac progression (6 months), hematologic VGPR (6 months), hematologic PR (the best) |
| Six features selected  (6 features) | weight loss, UA, E/e’ ratio, LV GLS hematologic CR (the best), cardiac remission (the best) |

Abbreviations are the same as those in Table 1.

Table S3. Comparison of treatment, response and outcome in training and test tests

|  | Training set  N = 106 | Test set  N = 26 | *P* value |
| --- | --- | --- | --- |
| Status, nonsurvivors | 67 (63.2) | 16 (61.5) | 1 |
| Follow-up time, d | 435.00 (97.75, 1581.50) | 501.00 (64.00, 1780.75) | 0.918 |
| **Hematological medication** |  |  |  |
| ASCT | 1 (0.9) | 2 (7.7) | 0.099 |
| Bortezomib based | 64 (60.4) | 15 (57.7) | 0.978 |
| Daratumumab based | 12 (11.3) | 4 (15.4) | 0.519 |
| IMiDs based | 12 (11.3) | 4 (15.4) | 0.519 |
| Others | 15 (14.2) | 4 (15.4) | 1 |
| **Cardiac medication** |  |  |  |
| ACEI/ARB | 2 (1.9) | 1 (3.8) | 0.485 |
| Beta-blocker | 14 (13.2) | 4 (15.4) | 0.755 |
| CCB | 6 (5.7) | 0 (0.0) | 0.598 |
| Diuretic | 89 (84.0) | 21 (80.8) | 0.77 |
| **Hematologic response** |  |  |  |
| CR |  |  |  |
| 1 month | 16 (15.1) | 2 (7.7) | 0.524 |
| 3 months | 26 (24.5) | 4 (15.4) | 0.462 |
| 6 months | 38 (35.8) | 5 (19.2) | 0.166 |
| Best response | 49 (46.2) | 6 (23.1) | 0.054 |
| VGPR |  |  |  |
| 1 month | 0 (0.0) | 1 (3.8) | 0.197 |
| 3 months | 2 (1.9) | 0 (0.0) | 1 |
| 6 months | 6 (5.7) | 0 (0.0) | 0.598 |
| Best response | 10 (9.4) | 2 (7.7) | 1 |
| PR |  |  |  |
| 1 month | 17 (16.0) | 3 (11.5) | 0.763 |
| 3 months | 18 (17.0) | 2 (7.7) | 0.362 |
| 6 months | 9 (8.5) | 1 (3.8) | 0.686 |
| Best response | 10 (9.4) | 2 (7.7) | 1 |
| No |  |  |  |
| 1 month | 15 (14.2) | 4 (15.4) | 1 |
| 3 months | 10 (9.4) | 2 (7.7) | 1 |
| 6 months | 7 (6.6) | 1 (3.8) | 1 |
| **Cardiac response** |  |  |  |
| Remission |  |  |  |
| 1 month | 11 (10.4) | 2 (7.7) | 1 |
| 3 months | 19 (17.9) | 3 (11.5) | 0.565 |
| 6 months | 29 (27.4) | 4 (15.4) | 0.312 |
| Best response | 45 (42.5) | 6 (23.1) | 0.111 |
| Progression |  |  |  |
| 1 month | 30 (28.3) | 4 (15.4) | 0.272 |
| 3 months | 27 (25.5) | 2 (7.7) | 0.09 |
| 6 months | 21 (19.8) | 5 (19.2) | 1 |

Abbreviations are the same as those in Table 1.
