# Supplementary figures and images for: Beyond NT-proBNP and troponin: How machine learning redefines light-chain cardiac amyloidosis risk assessment
Source: BMC Med Inform Decis Mak. 2025 Oct 9;25:367. doi: 10.1186/s12911-025-03207-0 (PMC12512860; doi:10.1186/s12911-025-03207-0)

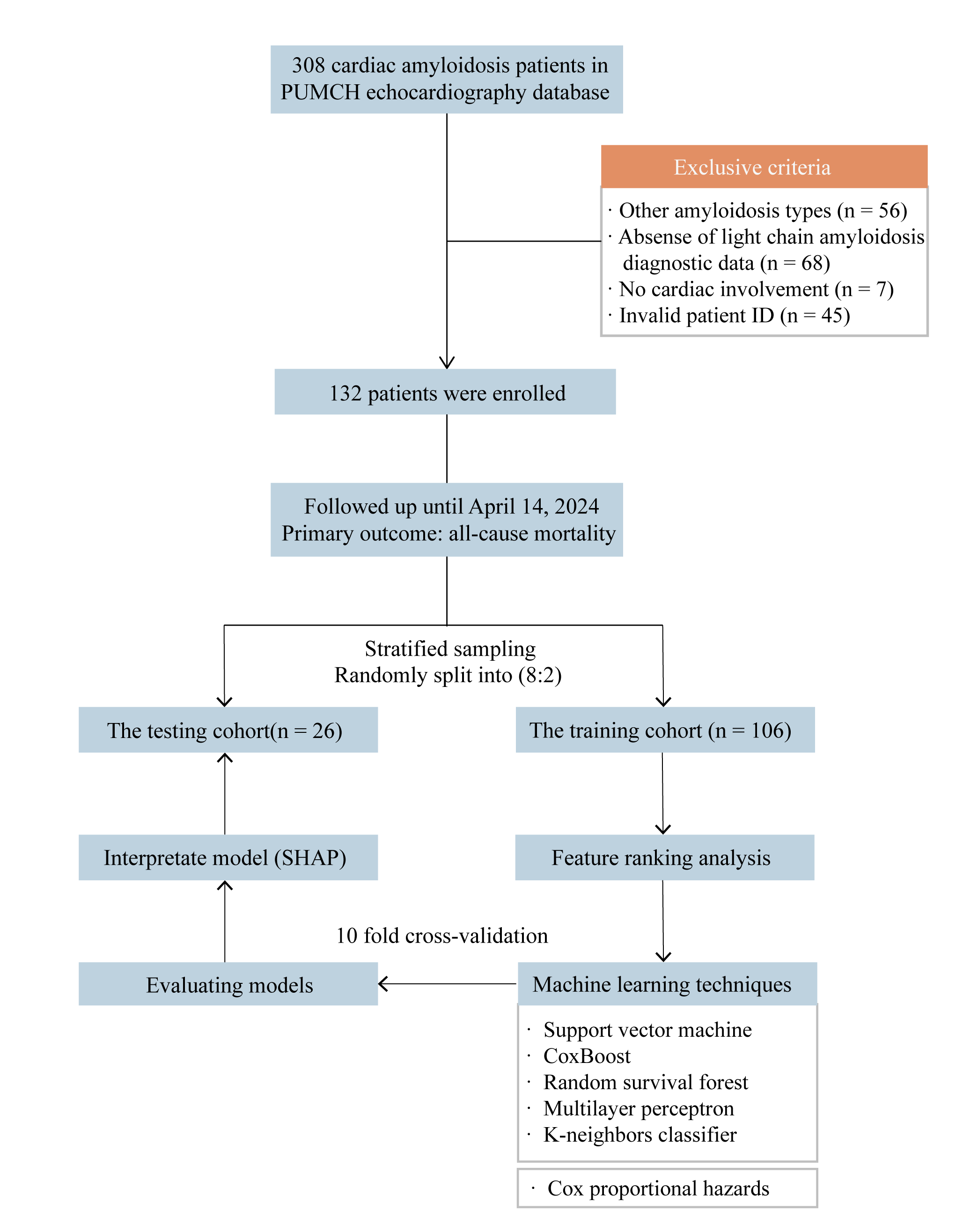

Supplement: Supplementary file 2 — Supplementary Material 2 [file 12911_2025_3207_MOESM2_ESM.tif]

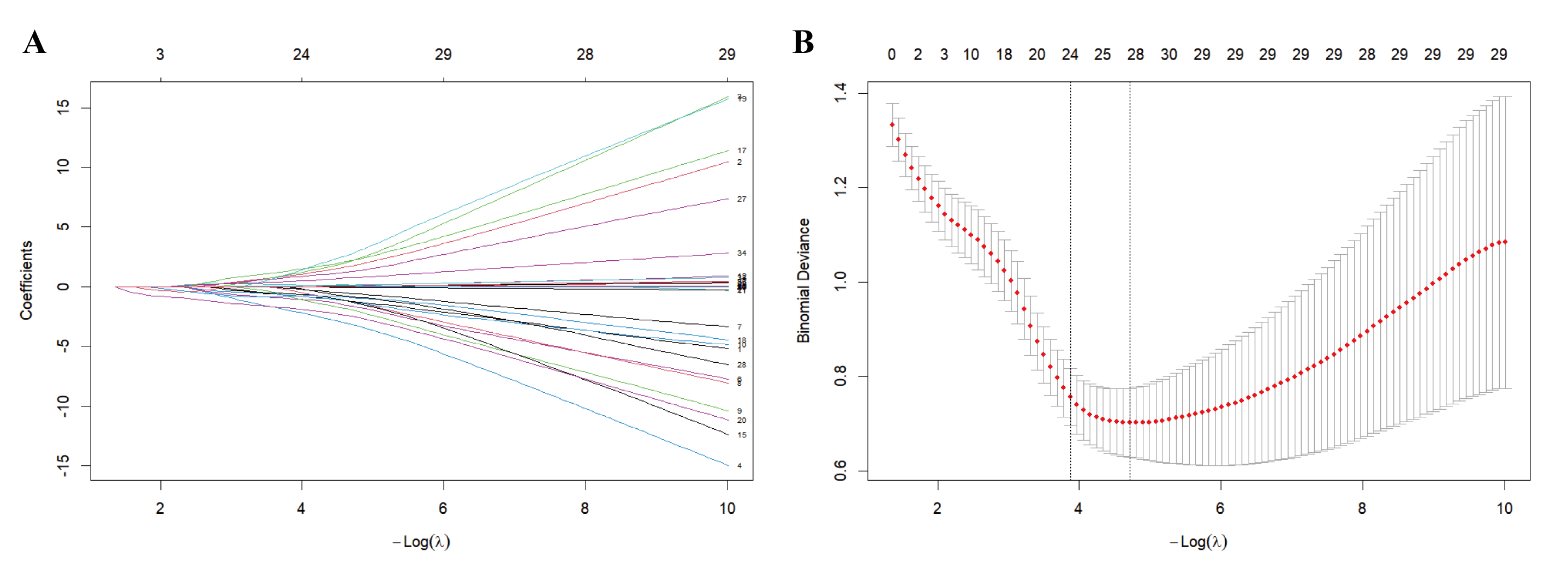

Supplement: Supplementary file 3 — Supplementary Material 3 [file 12911_2025_3207_MOESM3_ESM.tif]

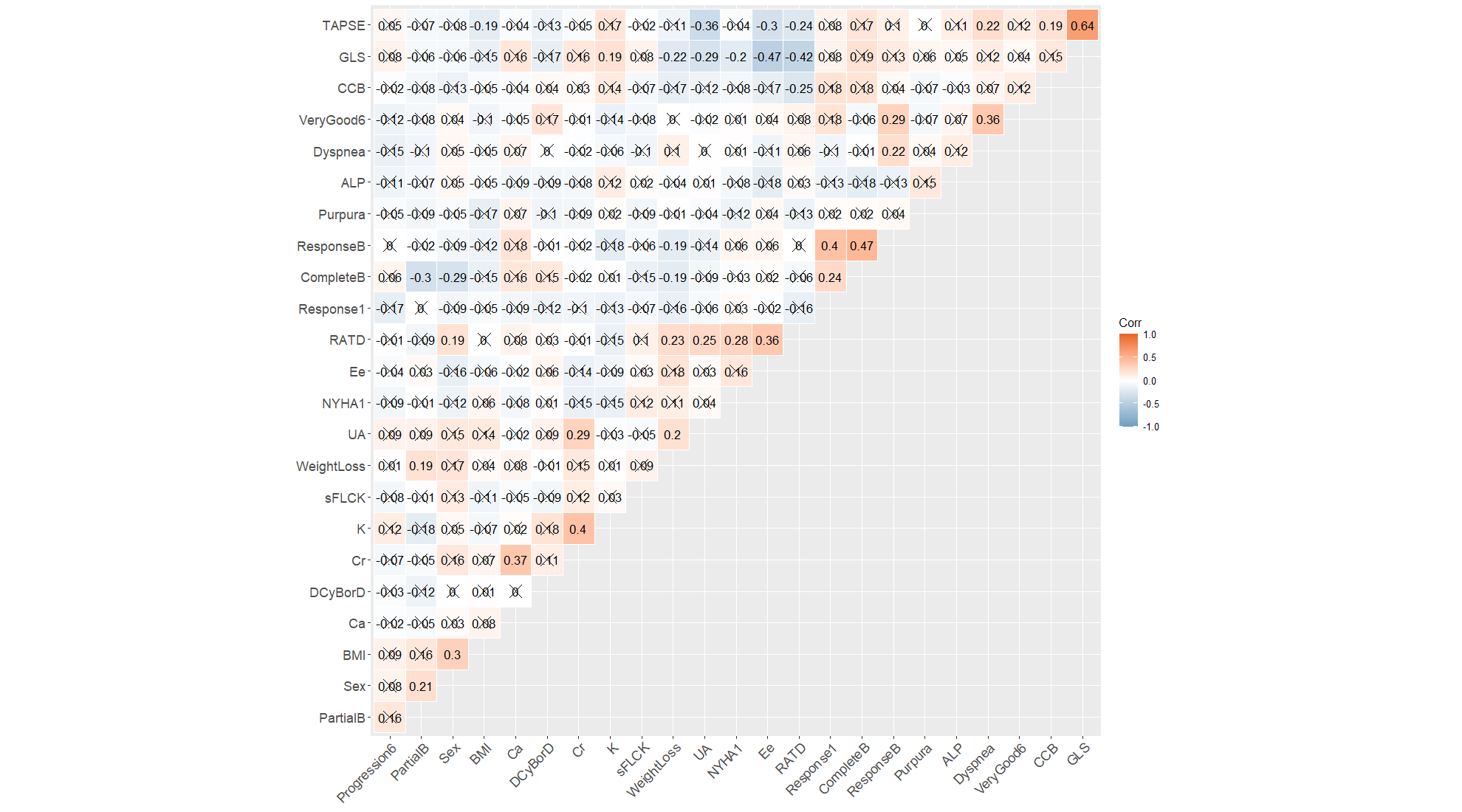

Supplement: Supplementary file 4 — Supplementary Material 4 [file 12911_2025_3207_MOESM4_ESM.tiff]

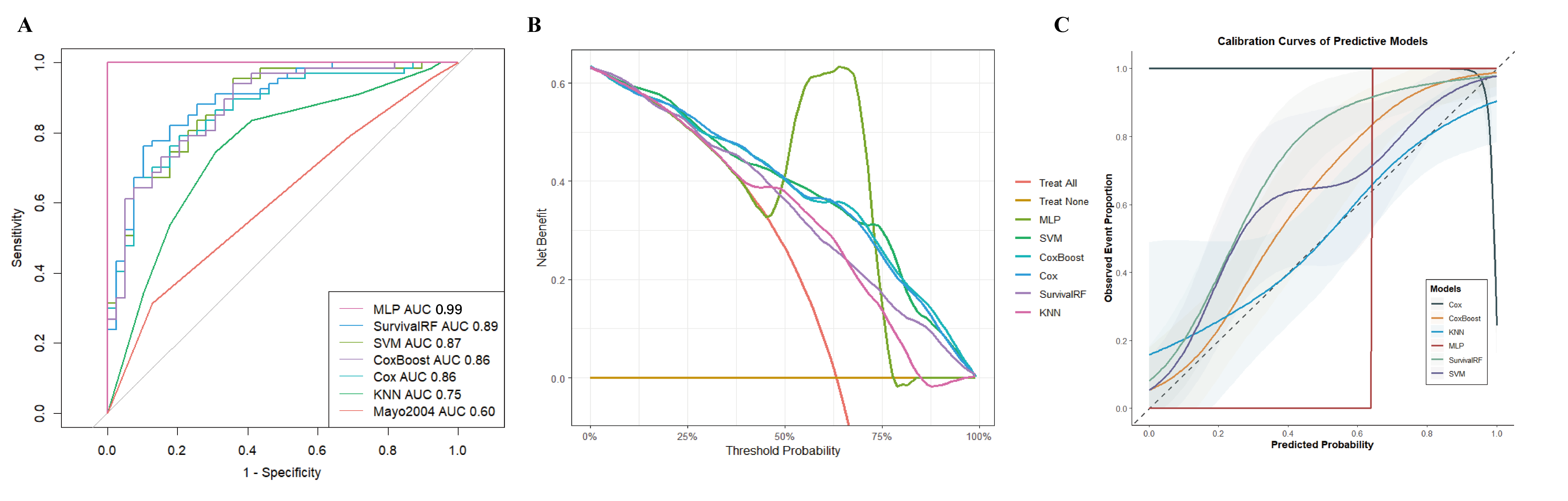

Supplement: Supplementary file 5 — Supplementary Material 5 [file 12911_2025_3207_MOESM5_ESM.tif]
